# Income inequality, gene expression, and brain maturation during adolescence

**DOI:** 10.1038/s41598-017-07735-2

**Published:** 2017-08-07

**Authors:** Nadine Parker, Angelita Pui-Yee Wong, Gabriel Leonard, Michel Perron, Bruce Pike, Louis Richer, Suzanne Veillette, Zdenka Pausova, Tomas Paus

**Affiliations:** 1 Institute of Medical Science, University of Toronto, Toronto, Canada; 2 Rotman Research Institute, University of Toronto, Toronto, Canada; 3 Department of Psychology, University of Toronto, Toronto, Canada; 4 Montreal Neurological Institute, McGill University, Montreal, Canada; 5 ECOBES, Cégep de Jonquière, Jonquiere, Canada; 6 University of Quebec in Chicoutimi, Chicoutimi, Canada; 7 Department of Radiology and Hotchkiss Brain Institute, University of Calgary, Calgary, Canada; 8 Hospital for Sick Children, University of Toronto, Toronto, Canada; 9 Department of Psychiatry, University of Toronto, Toronto, Canada; 10 Child Mind Institute, New York, United States

## Abstract

Income inequality is associated with poor health and social outcomes. Negative social comparisons and competition may involve the hypothalamic-pituitary-adrenal (HPA) and hypothalamic-pituitary-gonadal (HPG) axes in underlying some of these complex inter-relationships. Here we investigate brain maturation, indexed by age-related decreases in cortical thickness, in adolescents living in neighborhoods with differing levels of income inequality and household income. We examine whether inter-regional variations relate to those in glucocorticoid receptor (HPA) and androgen receptor (HPG) gene expression. For each sex, we used a median split of income inequality and household income (income-to-needs ratio) to create four subgroups. In female adolescents, the high-inequality low-income group displayed the greatest age-related decreases in cortical thickness. In this group, expression of glucocorticoid and androgen receptor genes explained the most variance in these age-related decreases in thickness across the cortex. We speculate that female adolescents living in high-inequality neighborhoods and low-income households may experience greater HPA and HPG activity, leading to steeper decreases in cortical thickness with age.

## Introduction

The rising disparity in personal incomes among people living in the same geographic unit (e.g., country or state) has led to a surge in research concerning the negative impact of this phenomenon on health outcomes. Higher levels of income inequality have been linked to higher rates of mortality and morbidity, the latter including obesity, cardiovascular disease and mental illness^1–6^. Using survey data from 12 developed countries, Pickett and Wilkinson (2010) discovered that countries with higher income-inequality (e.g., USA or UK) had three times as many individuals with mental illness than those with lower income-inequality (e.g., Italy or Germany)^5^. The nature of the relationship between income inequality and population health is unclear. One theory, the “Income Inequality Hypothesis”, maintains that health is impacted directly by income inequality within a society^7–9^. Two versions of this hypothesis have been put forward. The “strong” version states that high levels of income inequality impacts health of *all* individuals within a society^10^. The “weak” version proposes that large inequalities in income produce more pronounced negative health outcomes in poorer (vs. richer) members of society^8, 10^. The weak version is supported by a Kahn *et al*. (2000) study, which found higher rates of depressive symptoms and poorer self-rated health in mothers at the bottom 20% of household income in the sample who also lived in states with higher income inequality^11^.

The psychosocial interpretation is one of the dominant views on how income inequality may influence population health. From this view, psychosocial stress generated by individual’s perception of his/her position within the socioeconomic hierarchy mediates the negative health consequences of income inequality^9, 12, 13^. Such social comparisons relate to less social cohesion and higher rates of mistrust within a society that may, in turn, increase individual levels of stress and anxiety^14^. Social comparisons may also breed competition and aggression; societies with greater inequality also have higher rates of violent crimes^15–17^. From a physiological standpoint, chronic stress and aggression are associated with higher circulating levels of cortisol and testosterone, respectively^18, 19^. Both cortisol and testosterone rise in concert in response to threats to social status^19^.

Within the social environment of adolescents, peer comparisons and social stratification breeds competition; achieving and maintaining a favorable social status becomes crucial^20–22^. At the same time, the brain continues to develop; among various metrics, cortical thickness and volume decrease from childhood through adolescence into early adulthood^23–26^. Testosterone and cortisol appear to play a role in such age-related cortical thinning during this developmental period^27–30^. These social, hormonal, and neurodevelopmental factors may help to explain the onset of some psychiatric disorders, such as depression and schizophrenia during adolescence and early adulthood^27, 31, 32^. With the unique characteristics of social development and neural development during adolescence, this period may be particularly sensitive with regards to the potential influence of income inequality on brain development.

Several previous studies have addressed associations between socio-economic parameters, such as family income and parental education, and cognitive development (reviewed by Hackman and Farah 2009; Hackman *et al*.^33–35^). Only a handful of investigations studied variations in brain structure during development as a function of socioeconomic status (SES)^35–38^. For example, lower SES appears to be related to smaller (overall) cortical surface and thinner prefrontal cortex^36, 37^. Additionally, Piccolo *et al*. (2016) observed that both family income and parental education moderate non-linear age-related variations in cortical thickness. To our knowledge, there has been no investigation into the relationship between income *inequality* and brain development.

The aim of the current study is to test for possible associations between income inequality and brain maturation, the latter indexed by age-related decreases in cortical thickness during adolescence. First, we investigate inter-individual variations in age-related decreases in cortical thickness as a function of income inequality; based on the weak income-inequality hypothesis, we split our sample by median household-income (low, high). In view of the known sex differences in cortical maturation during adolescence^39–42^, we carry out these investigations separately in female and male adolescents. Next, to explore possible roles of cortisol and testosterone in the relationship between income inequality and brain maturation, we relate inter-regional variations in age-related decreases in cortical thickness to those in the expression of glucocorticoid receptor (*NR3C1*) and androgen receptor (*AR*) genes across the human cerebral cortex. Given the association between inequality and social competition, we predicted that these inter-regional relationships between decreases in thickness and gene expression should be stronger in the group with high income-inequality and low household-income.

## Results

This investigation used data from the Saguenay Youth Study of 1029 adolescents from the Saguenay Lac Saint Jean Region of Quebec Canada. Participants with incomplete household income and number of household person’s information (n = 11), and missing or unusable imaging data (n = 33) were excluded. We used Canadian Census data at the level of the census tract as our neighborhood equivalent; therefore, participants living in non-census tract locations (n = 181) were also excluded. This left 804 adolescents included in analysis, of which 404 were female (Age: mean = 179.93 ± 22.37 months) and 400 males (Age: mean = 179.07 ± 21.74 months).

For each sex, we created four subgroups based on income inequality and household income. The Gini coefficient, a common measure of income inequality, was calculated using Canadian Census data on the distribution of household income within each of the 37 census tracts (neighborhoods). To account for influence of overall neighborhood wealth, census-tract mean household income (from Canadian Census) was regressed against tract Gini coefficient. The resulting (Gini) residuals were split by median into high (n = 18, mean ± SD Gini: 0.46 ± 0.03) and low (n = 19, mean ± SD Gini: 0.41 ± 0.03) income-inequality tracts. Inequality (Gini coefficient) in the 37 census tracts ranged between 0.34 and 0.56. The distribution of Gini coefficients can be seen in Supplementary Figure 1. Participants were divided into income groups based on an annual household income reported by the parents during the study. We calculated an income-to-needs ratio by dividing household income reported by the participants by the low-income cut-offs reported by Statistics Canada for the same family size. Next, a median split of this adjusted household-income was performed to create high-income and low-income groups. These income-inequality (inequality) and household-income (income) groupings yielded four subgroups per sex: Low-income High-inequality, Low-income Low-inequality, High-income High-inequality, and High-income Low-inequality. Demographic, socioeconomic and physical characteristics of these sex-stratified subgroups can be found in Supplementary Table 1.

### Neighborhood Characteristics

Socioeconomic and housing information was collected from the 2006 Canadian census and analyzed to determine any differences between high and low income-inequality neighborhoods. As shown in Table 1, high income-inequality was associated with greater population density, larger proportion of apartment living (vs. houses), and less home ownership.

**Table 1:** Low Vs High Inequality Census Tract Demographics and Housing*.

Variables	Low Inequality	High Inequality	p-value
Number of Census Tracts	19	18	
Mean Gini (range)	0.41 ± 0.03	0.46 ± 0.03	1.13E-10
Census Tract Mean Income	57654.84 ± 8106.7	53204.83 ± 14510.3	0.15
Population Size	4036.4 ± 16131.0	4163.9 ± 1540.66	1.00
Population Density (/km^2^)	742.76 ± 983.36	1503.47 ± 1017.64	0.01
**Education** (**%**)			
Did Not Complete High School	14.87	15.04	0.99
University Degree	17.75	23.76	0.09
**Housing Types** (**%**)			
Single Detached Houses	59.59	38.00	5.36E-04
Semi-Detatched Houses	6.28	6.94	1.00
Row houses	2.12	2.31	0.52
Other Single Attached House	0.34	0.47	0.78
Apartment Duplex	13.67	15.32	0.28
Apartment (<5 stories)	15.72	33.73	1.51E-04
Apartment (≥5 stories)	0.04	3.13	4.62E-03
Movable Dwelling	2.24	0.11	0.01
**Condition of Dwelling** (**%**)			
Regular House Maintenance Only	67.64	69.80	0.48
Minor House Repairs	25.49	23.63	0.30
Major House Repairs	6.86	6.56	0.60
**Types of Occupants** (**%**)**			
Occupied by Owner	71.48	55.02	0.02
Occupied by Renter	28.52	44.98	

*All data was obtained from the 2006 Canadian Census. P-values were generated using non-parametric Man-Whitney test unless otherwise specified. **p-value generated by chi-squared test.

### Income Inequality and the Relationship between Cortical Thickness and Age

**Figure 1:**
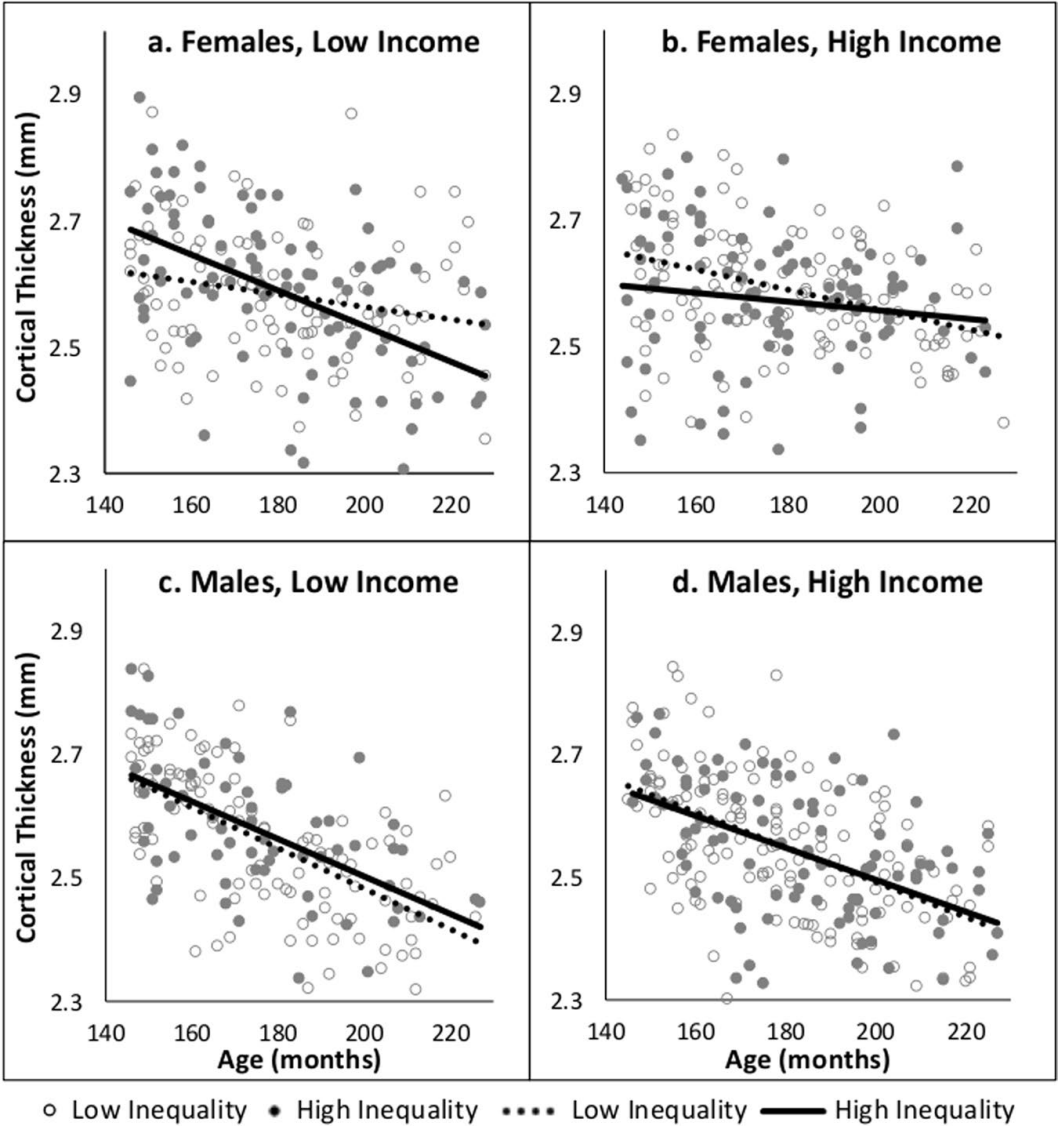
Mean cortical thickness by age in male and female income (low, high) groups. Within each income group plot inequality subgroups are stratified: high = solid circles and solid line, low = hollow circles and dashed line. In predicting cortical thickness, the interaction between age by inequality was analyzed for each male and female income group.

Mean (overall) cortical thickness for each cerebral hemisphere was generated by FreeSurfer software (version 5.3) and averaged across the two hemispheres for a single value for each participant. The relationship between cortical thickness and age varied by sex (Age-by-Sex interaction: F(1,802) = 20.18, p < 0.0001; females: R^2^ = 0.10, p < 0.0001; males: R^2^ = 0.31, p < 0.0001). Therefore, males and females were analyzed separately. Using hierarchical linear models (for adolescents nested in families), we investigated the effect of age (months), income (high vs low), inequality (high vs low) and their interactions on cortical thickness. A three-way interaction of Age-by-Income-by-Inequality was observed in females (F(1,402) = 8.70, p = 0.003) but not males (F(1,398) = 0.12, p = 0.73). We followed up this interaction model by examining low-income and high-income groups separately in males and females. Figure 1 illustrates the relationship between cortical thickness and age for each income group and their inequality subgroups (high vs low), shown separately for females and males. An Age-by-Inequality interaction was observed for females in the low income (F(1,189) = 6.73, p = 0.029) but not the high income group F(1,211) = 1.85, p = 0.294). As seen in Fig. 1, for low-income females, high inequality (solid line) was associated with stronger negative correlation of thickness with age than low inequality (dotted line) (High Inequality: R^2^ = 0.255, p < 0.0001; Low Inequality: R^2^ = 0.050, p = 0.029). This was not the case for high-income females (High Inequality: R^2^ = 0.017, p = 0.20; Low Inequality: R^2^ = 0.14, p < 0.0001). For males, no Age-by-Inequality interaction was observed in the low (F(1,173) = 0.70, p = 0.41) or high (F(1,223) = 0.001, p = 0.97) income group. Finally, we conducted pair-wise slope comparisons of each subgroup. Females of the Low-income High-inequality group had the steepest decline in cortical thickness with age (Low-income High-inequality (R^2^ = 0.26) > Low-Income Low-inequality (R^2^ = 0.05), p = 0.005; Low-income High-inequality (R^2^ = 0.26) > High-Income Low-inequality (R^2^ = 0.14), p = 0.02; Low-income High-inequality (R^2^ = 0.26) > High-Income High-inequality (R^2^ = 0.02), p = 0.002). All male subgroups had similar strong associations between age and cortical thickness (Fig. 1). Note that adding the quadratic term for age in all models did not change the above results.

To allow for a comparison with previous studies of cortical thickness vis-à-vis family income^35^ and general intelligence^43^, we have re plotted age-related decreases in cortical thickness as a function of these two variables (Supplemental Information).

### Inter-regional variations in *NR3C1* and *AR* Expression and Decreases in Cortical Thickness

To investigate inter-regional variations in age-related decreases in cortical thickness in relation to inter-regional variations in gene expression, the cerebral cortex was parcellated using FreeSurfer software into 34 regions per hemisphere; mean thickness for each region was averaged across the left and right hemisphere. Regional correlations between cortical thickness and age were performed. Correlation coefficients were Fisher z-transformed and used as dependent variables in the subsequent analyses testing the relationships between age-related variations in thickness and gene expression *across* the 34 regions. For each region, we used gene expression values for glucocorticoid receptor (*NR3C1*) and androgen receptor (*AR*) generated from the Allen Brain Atlas^44, 45^.

**Figure 2:**
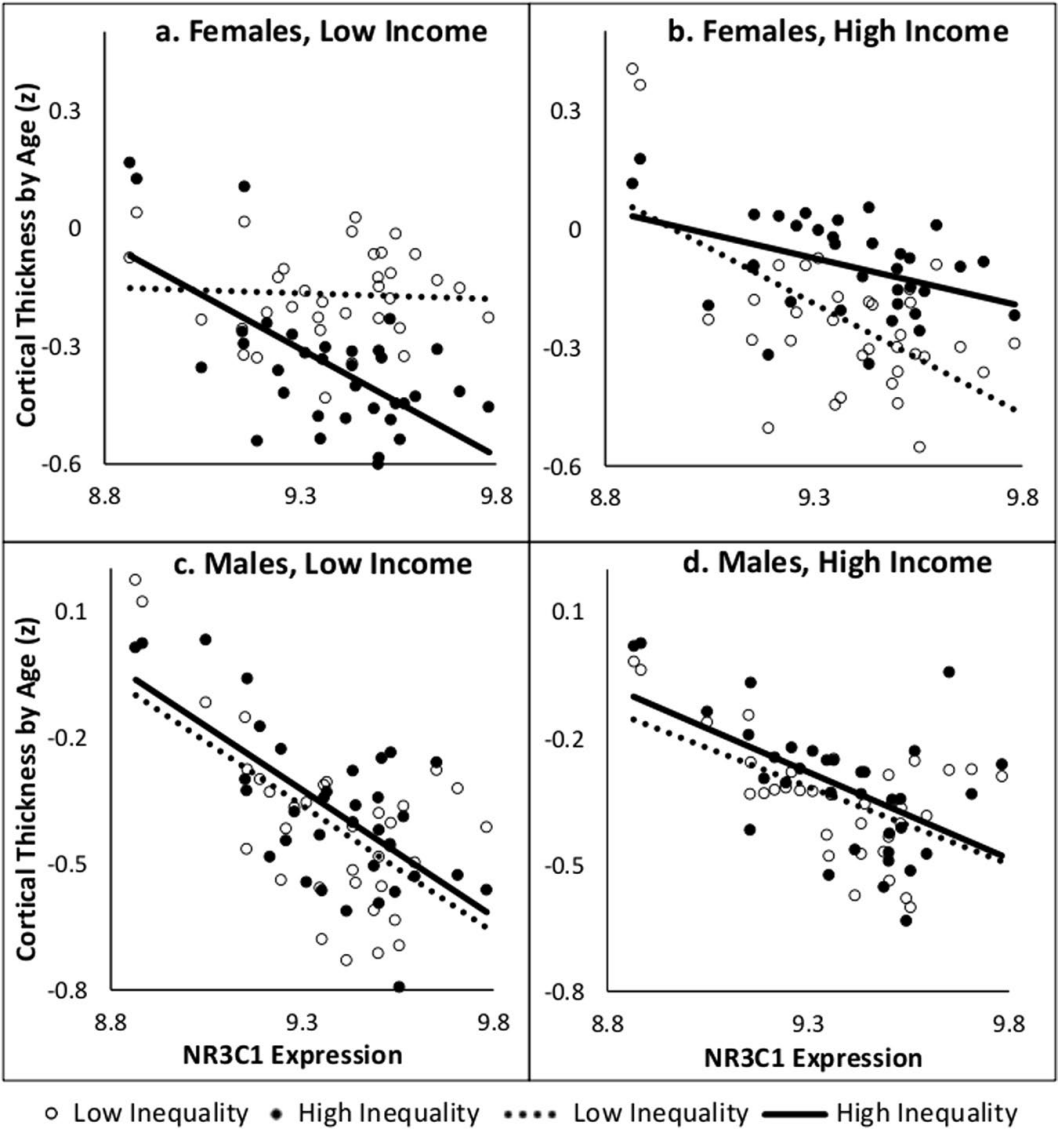
The y-axis (Cortical Thickness by Age) is the Fisher z-transformed correlation coefficients of cortical thickness and age in each of the 34 regions. The x-axis is the *NR3C1* expression in each of these regions. For each subgroup, there are 34 points representing each cortical region. Each male and female income subgroups are divided into high (solid circle, solid line) and low (hollow circle, dashed line) inequality subgroups. An inequality by NR3C1 expression interaction was analyzed for each income group.

We tested for a three-way interaction between *NR3C1* expression, income, and inequality within each sex. Fisher Z-transformed correlation coefficients (cortical thickness by age) for the 34 cortical regions were used as dependent variables. A three-way interaction of Expression-by-Income-by-Inequality was present for females (F(1,134) = 14.33, p = 0.0002) but not males (F(1,134) = 0.04, p = 0.85). Figure 2 illustrates the relationship of these variables for both male and female subgroups. Names and gene-expression levels for each of the 34 cortical regions can be found in Supplementary Table 2. We then followed up the interaction model by testing the relationship between inter-regional variations in age-related decreases in cortical thickness and those in *NR3C1* expression in low- and high-income groups for both sexes. Females from low-income families showed an interaction between *NR3C1* expression and inequality (F(1,66) = 11.78, p = 0.001); as seen in Fig. 2, high-inequality females had a steeper negative association compared with low-inequality females (p = 0.0011). In the female high-income group, we observed no interaction between *NR3C1* expression and inequality (F(1,66) = 3.88, p = 0.053). For males, no Expression-by-Inequality interaction was observed for low-income (F(1,66) = 0.16, p = 0.69) or high-income (F(1,66) = 0.10, p = 0.76) groups (Fig. 2). Finally, we analyzed the association between *NR3C1* and age-related decreases in cortical thickness in each subgroup. Females in the low-income high-inequality subgroup displayed the strongest association between decreases cortical thickness with age and *NR3C1* expression (Low-income High-inequality: R^2^ = 0.41, p < 0.0001; Low-Income Low-inequality: R^2^ = 0.003, p = 0.76; High-Income Low-inequality: R^2^ = 0.36, p = 0.0002; High-Income High-inequality: R^2^ = 0.18, p = 0.01). For males, associations between inter-regional variations in age-related decreases in cortical thickness and *NR3C1* expression were present in all four subgroups (Low-income High-inequality: R^2^ = 0.45, p < 0.0001; Low-Income Low-inequality: R^2^ = 0.38, p < 0.0001; High-Income Low-inequality: R^2^ = 0.33, p = 0.0003; High-Income High-inequality: R^2^ = 0.30, p = 0.0007).

**Figure 3:**
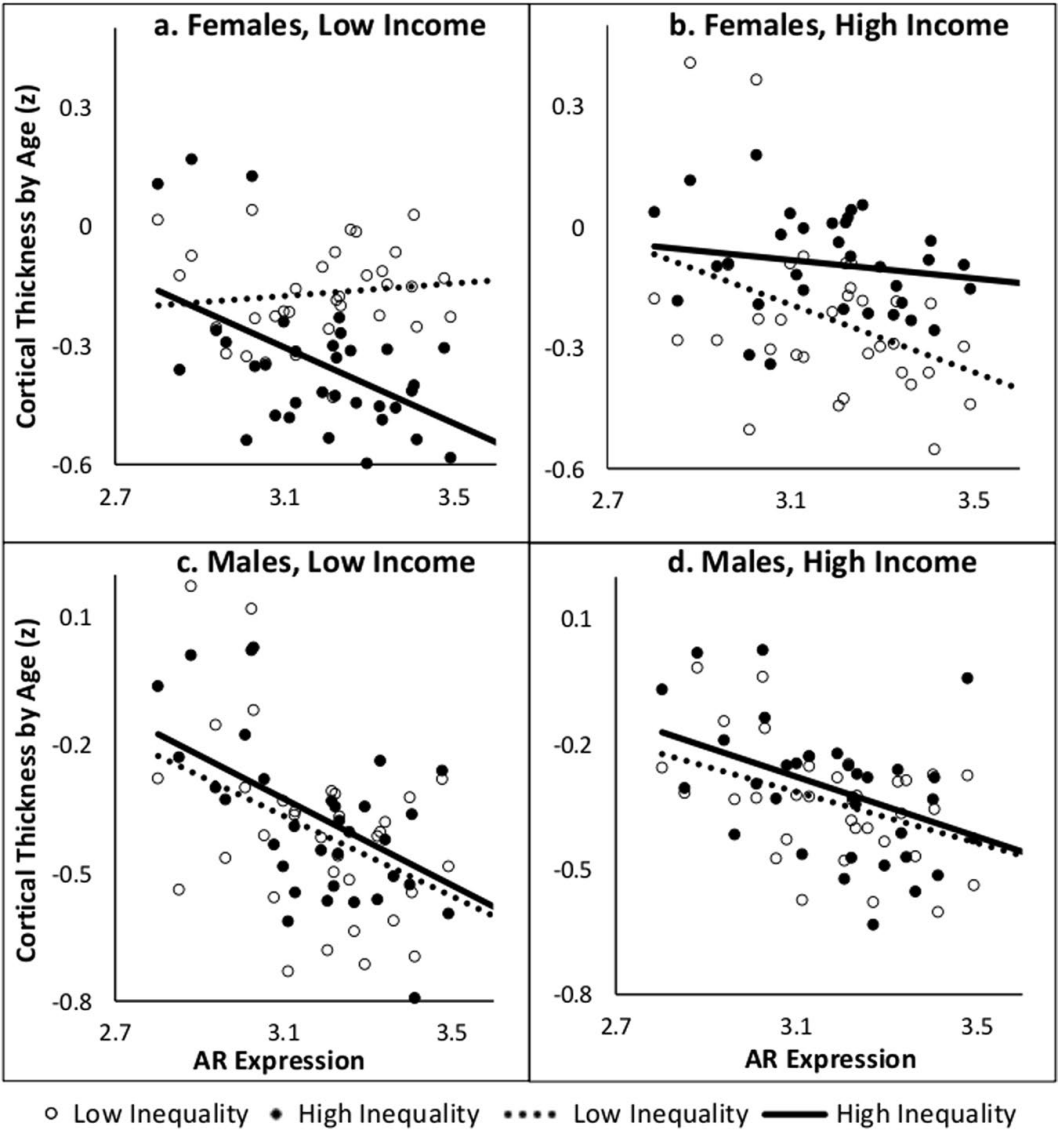
The y-axis (Cortical Thickness by Age) is the Fisher z-transformed correlation coefficients of cortical thickness and age in each of the 34 regions. The x-axis is the *AR* expression in each of these regions. For each subgroup, there are 34 points representing each cortical region. Each male and female income subgroups are divided into high (solid circle, solid line) and low (hollow circle, dashed line) inequality subgroups. An inequality by AR expression interaction was analyzed for each income group.

Expression levels of *AR* and *NR3C1* across these 34 cortical regions were correlated (r = 0.75, p < 0.0001). Therefore, it is not surprising that we observed a similar pattern of results for *AR* expression. We examined associations between age-related decreases in cortical thickness and *AR* expression across the 34 cortical regions in a three-way interaction between *AR* expression, income and inequality. This interaction was present in females (F(1,134) = 10.42, p = 0.002) but not males (F(1,134) = 0.003 p = 0.95). As shown in Fig. 3, stratifying by income group revealed a two-way interaction between *AR* expression and inequality in low-income females (F(1,66) = 9.78, p = 0.003); this was not the case for high-income females (F(1,66) = 2.35, p = 0.13). Again, males did not show any Expression-by-Inequality interactions in either income group (Low Income: F(1,66) = 0.03, p = 0.87; High Income: F(1,66) = 0.09, p = 0.76). Finally, we analyzed the association between *AR* and decreases in cortical thickness in each subgroup. Females in the low-income high-inequality subgroup displayed the strongest association between age-related decreases in cortical thickness and gene expression across the 34 regions (Low-income High-inequality: R^2^ = 0.26, p = 0.002; Low-Income Low-inequality: R^2^ = 0.02, p = 0.46; High-Income Low-inequality: R^2^ = 0.17, p = 0.02; High-Income High-inequality: R^2^ = 0.03, p = 0.31). For males, associations between decreases in cortical thickness and *AR* expression were present in all four subgroups (Low-income High-inequality: R^2^ = 0.26, p = 0.002; Low-Income Low-inequality: R^2^ = 0.19, p = 0.01; High-Income Low-inequality: R^2^ = 0.19, p = 0.01; High-Income High-inequality: R^2^ = 0.19, p = 0.01).

### Cortical Thickness Adjustments: Accounting for Subgroup Differences

With the exception of parental education and maternal smoking during pregnancy, we observed no differences between high and low inequality groups (stratified by income) in demographics, SES, or a number of physical characteristics (Supplementary Table 1). For this reason, cortical thickness was adjusted for parental education and maternal smoking during pregnancy. We also included the age-squared term and interactions (Age^2^-by-Income-by-Inequality, Age^2^-by-Income). Using the adjusted cortical thickness, all interactions and slope differences described above using unadjusted data remained virtually unchanged (See Supplementary Tables 2 and 3).

## Discussion

As demonstrated previously, cortical thickness decreases with age in both male and female adolescents. Also, consistent with previous studies, age explained greater variance in cortical thickness in males than in females^39, 42, 46^. Here we have discovered that the age-related decreases in cortical thickness appear to be steeper in female adolescents living in neighborhoods with high inequality *and* households with low income. This group of female adolescents showed the steeper decline in cortical thickness in comparison with the other same-sex subgroups. We then tested the possible role of stress and sex hormones in explaining this phenomenon. We found a strong association between inter-regional variations in age-related decreases in cortical thickness and expression of both *NR3C1* and *AR* across the 34 regions in the same group of female adolescents (i.e. high-inequality low-income). These results illustrate a potential influence of social environment, namely living in low–income households located in high-inequality neighborhoods, on maturation of the adolescent brain.

High-inequality areas in the studied region displayed higher population density than low inequality areas. Threats to social status and social stress are elevated in high population density urban-areas, and these environments are related to higher rates of mental illness^47–49^. To some extent, the differences in population density may contribute to the observed pattern of relationships between inter-regional variations in age-related decreases in cortical thickness and the expression of glucocorticoid- and androgen-receptor genes in low-income females. The interplay between one’s social environment and the neural processing of social stressors was examined in a study by Lederbergen *et al*.^50^. The study used functional MRI to show that individuals from urban upbringing and those currently living in cities had higher response to social stressors in the anterior cingulate cortex and the amygdala, as compared with those growing up (or living) in rural areas.

In addition to urbanicity, low household income and high income-inequality have been associated with greater stress and mental illness^12, 13, 51, 52^. Exposure to environmental stressors coupled with increased sensitivity to stress in adolescent females^53^ may explain the observed steeper decreases in cortical thickness with age seen in the low-income high-inequality female group. At this point, we can only speculate whether or not this apparent difference in cortical maturation increases the risk for mental illness later in life. Given the rich literature on lower cortical thickness in a number of psychiatric disorders^54–56^, as compared with healthy controls, this possibility should be tested in future longitudinal studies. Such studies may clarify under what conditions higher (or lower) rate of cortical thinning represents an index of a negative (or positive) developmental process. Without stratifying by sex (or income inequality), previous studies observed higher rates of cortical thinning in children and adolescents from high (vs. low) income families^35^, high (vs. low) general intelligence^43^, and in typically developing (vs. ADHD) individuals^57^. Elevated stress levels can impact cortical morphology by reducing the complexity of dendritic arborization, ultimately leading to reductions of cortical volume and thickness^27, 58^. As we show by investigating the regional variation in age-related decreases in cortical thickness, the underlying mechanisms to this steeper decrease may lie in the activation of glucocorticoid receptor. In a study of glucocorticoid receptor mRNA levels across the life span, higher expression levels were found in the cerebral cortex of adolescents and adults, as compared with both infants and older adults (age > 68)^59^.

Activation of the hypothalamic-pituitary-gonadal (HPG) axis during adolescence shapes brain development; testosterone plays a key role in this process^29, 60^. For example, Raznahan *et al*. (2010) found that female adolescents possessing alleles for a more efficient *AR* had greater cortical thinning in frontal cortical regions^40^. We suggest that a combined effect of stress and androgens may explain the observed phenomenon. The mechanisms involving the release of stress and sex hormones are complex and potentially interrelated. The hypothalamic-pituitary-adrenal (HPA) axis, involved in the stress response, and the HPG axis, involved in the release of sex hormones (e.g., testosterone), have been described as linked when responding to threats to social status and social evaluation^19^. In investigating the duality of the HPA and HPG axis in response to threats to social status, Turan *et al*. (2015) measured levels of salivary cortisol and testosterone in boys and girls (8–11 years) throughout a social stress task and found the two hormones to be elevated in concert^19^.

Given the combined influence of the two axes (HPG and HPA), and the presence of the elevated physiological response in both males and females, one would expect steeper decreases in cortical thickness with age in both sexes experiencing high levels of psychosocial stress. In our case, low-income adolescents living in high-inequality neighborhoods are the only study group exposed to upward comparisons of social status. These types of comparisons can be associated with negative self-evaluation and symptoms of depression and anxiety^61, 62^. But we observed steeper decreases in cortical thickness only in females (not males) living in this unique social environment. This may be due to a potential “ceiling effect” of testosterone. Testosterone levels are peaking in male adolescents^63^, possibly overshadowing any additional effects of cortisol associated with neighborhood inequality. Female adolescents may also be more responsive to stressors, leading to stress-related decreases in cortical thickness. For example, in a study of sex differences in depressive symptoms, women – as compared with men – reported experiencing more ruminating behavior, less feelings of mastery and a more frequent exposure to challenging life-situations^64^. In a multinational study of income inequality and life satisfaction in adolescence, income-inequality during the first 10 years of life predicted life dissatisfaction in female but not male adolescents^65^. These studies suggest females may be more susceptible to stressors associated with income inequality. We theorize that female participants from low-income homes and living in high-inequality neighborhoods have greater accumulation of stress with age (allostatic load) and, in turn, continue to show age-related decreases in cortical thickness into late adolescence. This theory is supported by our observation of a similarity between inter-regional profiles of these age-related decreases in cortical thickness and the inter-regional profile of *NR3C1* expression; this relationship was strong in the low-income high-inequality female adolescents

Our study has a number of strengths and weakness. Here, we measured income inequality at a small geographic level, namely a census tract. The majority of evidence in support of the relationship between income inequality and health, comes from studies carried out at the state/province and country levels (as reviewed by Wilkinson and Pickett)^13^. Nonetheless, a handful of studies assessed such relationships using smaller geographic units including census tracks employed here (addressed in a review by Wilkinson and Pickett)^66, 67^. The geographic level of analysis implicates different mechanisms by which income inequality might impact individual’s health and development. At country and state level, political/legislative elements may underlie relationships between income inequality and population health^66, 68^. At the neighborhood level, we suggest that psychosocial comparisons play a strong role. Furthermore, one can argue that measuring inequality across smaller vs. larger geographic units may decrease the range of inequality across the units. In our study, Gini coefficients range from 0.34 to 0.56. For comparison, Chen *et al*. (2012) measured income-inequality at county and state level, with Gini coefficients varying between 0.32 and 0.60 (county) and 0.40 and 0.54 (state). Ranges in Gini coefficients vary across studies, being both higher and lower than those reported here^65, 66, 68, 69^. Finally, we wish to point out that the key strength of our approach is that of relating aggregate data (income inequality) to *individual-level* data (brain maturation), thus overcoming common criticism of studies relating income inequality to health outcomes assessed at an aggregate level (e.g., prevalence of depression in a given geographic unit).

For obvious reasons, we do not have access to gene-expression levels in brains of adolescents participating in this study. This is why we relate variations in gene expression and age-related decreases in cortical thickness across cortical regions rather than across individuals. This approach has one possible limitation, namely representativeness of gene-expression data used for this purpose. For both *NR3C1* and *AR*, we used mean values of their expression in the human cerebral cortex as provided by the Allen Human Brain Atlas; these values were derived in a sample of six adult donors (one female). Are data derived from such a small sample representative of general population? We have addressed this issue in our previous study^30^ by comparing gene-expression profiles reported in the Allen Human Brain Atlas with those measured in another dataset, namely the BrainSpan Atlas (www.brainspan.org); in the latter, we used gene-expression data obtained in nine adolescents and young adults (13 to 40 years of age; five males, four females). Across 11 homologous cortical regions, we observed strong correlations in expression profiles between the two datasets for both genes (*NR3C1*: r = 0.82, *AR*: r = 0.86). This confirms a high level of representativeness of these inter-regional profiles in the expression of *NR3C1* and *AR* in the human cerebral cortex.

In conclusion, we set out to determine potential influences of neighborhood income-inequality on adolescent brain maturation, a previously unexplored topic. We identified an apparent high-risk group of females from low-income households living in high-inequality neighborhoods who experience steep decreases in cortical thickness during adolescence. We believe that dual activation of the HPA and HPG axis induced by living in households with low income embedded in high-inequality environment (i.e., in the vicinity of high-income households) may explain this phenomenon. These findings provide a link between income inequality and adolescent brain maturation with potential as a mechanistic relation to development of mental illness.

## Methods

### Participants

Participants were recruited and assessed in the context of the Saguenay Youth Study, a family-based study of adolescents born in the Metropolitan area of Saguenay, Quebec, Canada^70, 71^. This region has a population of approximately 156,305 and covers an area of 1,126 km^2^ with 3 main boroughs (Chicoutimi, Jonquiere and La Baie)^72, 73^. Study recruitment occurred between 2003–2012 when 1,029 adolescents (aged 12–18) underwent extensive assessment that included magnetic resonance imaging (MRI) of the brain and abdomen, cardio-metabolic and cognitive testing, and questionnaires on family environment, life habits, psychiatric symptoms, substance use, and personality^71, 74^. All participants included in the study are of single ethnicity, namely White Canadians of French decent. All methods were performed in accordance with the relevant guidelines and regulations. The study was approved by the Chicoutimi Hospital Research Ethic Committee. Written informed consent and assent were obtained from the parents and adolescents, respectively.

### Income inequality

To determine neighborhood-level income inequality, we used the 2006 Canadian census and obtained census-tract household income data for Saguenay (obtained through the University of Toronto subscription to the Canadian Census database). The 2006 Canadian Census falls in the middle of the study recruitment phase and offers the best estimation of neighborhood socioeconomic parameters at the time of testing. Statistics Canada defines a census tract as a small stable geographic area with a population of 2,500 to 8,000 with the stipulation that these areas be located within a Census Metropolitan Area^75^. Therefore, participants living outside such an area were excluded. The Saguenay “Census Metropolitan Area” is divided into 37 census tracts all of which were populated by study participants. Participants postal codes were mapped to a given census tract using a conversion file linking Canada Post postal codes with Statistics Canada geographic areas^76^. Next, we proceeded with calculating an index of income inequality, namely the Gini coefficient. The Gini coefficient is a widely used indicator of income inequality that ranges from a value of 0 to 1^77^. In a perfectly equal population where all individuals earn the same income, the Gini coefficient is 0. As a population becomes more unequal in income distribution, the Gini coefficient rises. If the total income in a population is possessed by a single person, the Gini coefficient is 1. Statistics Canada reports the distribution of household income in a census tract in 11 bins/ranges. For each census tract, the Gini coefficient was calculated from these bins of household income using the Binequality package in the program R^78^. To account for influence of overall neighborhood wealth, census-tract mean income (from Canadian Census) was regressed against tract Gini coefficient. The resulting (Gini) residuals were median split into high and low income-inequality tracts.

### Household Income

An income-to-needs ratio was calculated using Low Income Cut-offs (LICOs), a measure that takes into account household size and identifies families that “will likely devote a larger share of its income on the necessities of food, shelter and clothing than the average family” (http://www.statcan.gc.ca/pub/75f0002m/2012002/lico-sfr-eng.htm). The income-to-needs ratio was calculated by dividing household income reported by the participants by LICO of a family of the same size. To test the “weak” income-inequality hypothesis, a median split of the income-to-needs ratio was performed to create “high-income” and “low-income” groups.

### Measures of Socioeconomic and Physical Characteristics

During the study, we acquired detailed information about the participants, their parents and family circumstances (including maternal smoking during pregnancy). Parental education was divided into nine major categories ranging from non-completion of primary school to graduate-level education (see Supplementary Table 1).

Sexual maturation was assessed with the Puberty Development Scale, a questionnaire consisting of eight questions used to assess adolescent pubertal development and assign them to a particular Tanner stage^79^. There are five Tanner stages assessing physical development of secondary sex characteristics during adolescence^80, 81^. Additional socioeconomic and housing data were collected from the 2006 Canadian census for each of the 37 census tracts.

### Magnetic Resonance Imaging and Cortical Thickness

T1-weighted MRI images were acquired with a Phillips 1.0 T superconducting magnet using the following parameters: 3D RF-spoiled gradient echo scan with 140–160 sagittal slices 1mm isotropic resolution, TR = 25 ms, TE = 5 ms, and flip angle = 30°. This study used FreeSurfer version 5.3 for derivation of cortical thickness^82^. Cortical thickness was analyzed in two ways. First, average cortical thickness was calculated by averaging the FreeSurfer-derived mean cortical thickness in the left and right hemisphere. The second measure of cortical thickness involved parcellation of the cerebral cortex into 34 regions (per hemisphere) using the Desikan-Killiany Atlas^82^. Once parcellated, left and right hemisphere values were averaged for each of the 34 regions in each participant.

### Gene expression

Expression of the glucocorticoid receptor (*NR3C1*) and androgen receptor (*AR*) genes in the human cerebral cortex were obtained for each of the 34 cortical regions using expression data from the Allan Human Brain Atlas (six donors, left hemisphere). This procedure and its validity have been described in detail by French and Paus^45^.

### Statistical Analysis

The income-inequality (inequality) and household-income (income) groupings yielded four subgroups per sex: Low-income High-inequality, Low-income Low-inequality, High-income High-inequality, and High-income Low-inequality. These groups were compared with regards to: (1) age-related changes in cortical thickness; (2) similarity of inter-regional profiles in age-related decreases in cortical thickness and gene expression (*NR3C1*, *AR*). To test the “weak” income-inequality hypothesis, we compared low and high inequality groups within each income group.

All statistical analyses were conducted using R software (version 3.3.1). First, to investigate the association between cortical thickness and age, we tested for three-way interactions between age, income, and inequality. Significant interactions were investigated further by testing age and inequality interactions in high and low income groups separately. Pairwise comparisons of thickness-by-age slopes were run between each of the four subgroups. Second, we investigated whether inter-regional variations in age-related decreases in cortical thickness are similar to inter-regional variations in the expression of *NR3C1* and *AR* genes across the human cerebral cortex. For each of the 34 cortical brain regions, we correlated age and cortical thickness. Correlation coefficients were then Fisher z-transformed and used as dependent variables in testing the three-way interaction between income, inequality, and gene expression (*NR3C1* and *AR*). The Fisher z-transformation transforms correlation coefficients into normally distributed z values using the following formula:

$${\rm{z}}={0.5}^{\ast }[{\rm{l}}{\rm{n}}(1+{\rm{r}})\,-\,{\rm{l}}{\rm{n}}(1-{\rm{r}})]$$

Next, we tested for within-income group interactions between inequality and gene expression. In all cases false discovery rate corrected p-values with and alpha of 0.05 were used.

### Data Availability

Canadian Census data was available through the University of Toronto’s licensing agreement with the Computing in the Humanities and Social Sciences Data Centre (institution subscription needed).

Postal Code Conversion Files are provided by Statistics Canada’s Canadian Socio-economic Information Management System (institution subscription needed).

Gini Coefficient calculations for binned top coded income distributions were conducted using the “binequality” package in R reference manual available on CRAN (https://cran.r-project.org/web/packages/binequality/index.html).

Gene expression (*NR3C1* and *AR*) available through Allan Brain Atlas (http://human.brain-map.org). Acquisition of gene expression for FreeSurfer cortical regions using R (with available code) provided by French and Paus (2015) (https://www.ncbi.nlm.nih.gov/pmc/articles/PMC4584957/)^45^.

## Electronic supplementary material

Supplementary Information
