# Supplementary material for: Income inequality, gene expression, and brain maturation during adolescence
Source: Sci Rep. 2017 Aug 7;7:7397. doi: 10.1038/s41598-017-07735-2 (PMC5547165; doi:10.1038/s41598-017-07735-2)
Supplement: Supplementary file 1 — Supplementary Information [file 41598_2017_7735_MOESM1_ESM.doc]

**Income inequality, gene expression, and brain maturation during adolescence**

Nadine Parker^1,2^, Angelita Pui-Yee Wong^2,3^, Gabriel Leonard^4^, Michel Perron^5,6^, Bruce Pike^7^, Louis Richer^6^, Suzanne Veillette^5,6^, Zdenka Pausova^8^, *Tomas Paus^1,2,3,9,10^

**Supplementary Tables 1 – 4**

**Supplementary Figures 1 - 3**

**Supplementary Table 1: Subgroup Characteristics^1^**

| **Variable** | **Low-income High-inequality** | | **Low-income Low-inequality** | | **p-Value _(Low Income)_** | | **High-income High-inequality** | | **High-income Low-inequality** | | **p-Value _(High Income)_** | |  |
| --- | --- | --- | --- | --- | --- | --- | --- | --- | --- | --- | --- | --- | --- |
| **Females** | | | | | | | | | | | | |  |
| **Number of Participants** | 93 | | 98 | |  | | 94 | | 119 | |  | |  |
| **Demographics/SES** |  | |  | |  | |  | |  | |  | |  |
| Mean Age (range) | 181.0 ± 22.40  (146-228) | | 180.2 ± 22.44  (146-228) | | 0.72 | | 178.1 ± 21.05  (144-223) | | 179.6 ± 22.69  (145-227) | | 0.63 | |  |
| Mean Family Income^2^ | 35217.39  ± 15913.8 | | 36510.42  ± 13975.9 | | 0.56 | | 77234.04  ± 10565.1 | | 76848.74  ± 10039.5 | | 0.79 | |  |
| **Parental Education (%)^3^** | M | F | M | F | M | F | M | F | M | F | M | F |  |
| *Some primary school* | 2.17 | 0.00 | 0.00 | 0.00 | 0.05 | 0.03 | 1.06 | 1.06 | 0.00 | 0.00 | 0.11 | 0.01 |  |
| *Primary school* | 2.17 | 0.00 | 0.00 | 0.00 |  |  | 0.00 | 0.00 | 0.00 | 0.00 |  |  |  |
| *Some high school* | 19.6 | 26.1 | 21.9 | 19.8 |  |  | 3.19 | 1.06 | 8.40 | 8.40 |  |  |  |
| *High school* | 33.7 | 41.3 | 51.0 | 56.3 |  |  | 28.7 | 30.9 | 30.3 | 36.1 |  |  |  |
| *Some college* | 9.78 | 1.09 | 10.4 | 2.08 |  |  | 8.51 | 5.32 | 9.24 | 14.3 |  |  |  |
| *College* | 15.2 | 13.0 | 9.38 | 16.7 |  |  | 30.9 | 26.6 | 20.2 | 14.3 |  |  |  |
| *Some university* | 9.78 | 4.35 | 5.21 | 1.04 |  |  | 11.7 | 4.26 | 5.04 | 5.04 |  |  |  |
| *Bachelor’s degree* | 4.35 | 12.0 | 0.00 | 2.08 |  |  | 11.7 | 24.5 | 20.2 | 16.8 |  |  |  |
| *Graduate degree* | 3.26 | 2.17 | 2.08 | 2.08 |  |  | 4.26 | 6.38 | 6.72 | 5.04 |  |  |  |
| **Physical Characteristics** |  | |  | |  | |  | |  | |  | |  |
| Cortical Thickness | 2.59 ± 0.13 | | 2.58 ± 0.10 | | 0.84 | | 2.57 ± 0.11 | | 2.59 ± 0.10 | | 0.21 | |  |
| Pubertal Stage  (Tanner Scale) | 4.03 ± 0.80 | | 4.09 ± 0.67 | | 0.57 | | 3.99 ± 0.77 | | 4.07 ± 0.70 | | 0.45 | |  |
| Contraceptive Use^3^ (%) (yes/no) | 9.78 | | 12.6 | | 0.65 | | 18.1 | | 16.0 | | 0.72 | |  |
| Height (cm) | 159.90 ± 7.30 | | 159.86 ± 5.88 | | 0.96 | | 158.91 ± 7.57 | | 160.37 ± 6.85 | | 0.15 | |  |
| Weight (kg) | 58.22 ± 14.15 | | 57.77 ± 14.91 | | 0.83 | | 53.80 ± 11.02 | | 54.82 ± 11.59 | | 0.51 | |  |
| BMI | 22.66 ± 4.87 | | 22.52 ± 5.38 | | 0.86 | | 21.26 ± 3.90 | | 21.24 ± 4.02 | | 0.97 | |  |
| Fat Mass | 16.43 ± 9.15 | | 16.20 ± 10.25 | | 0.88 | | 13.00 ± 7.63 | | 13.44 ± 7.08 | | 0.67 | |  |
| Fat Free Mass  (Lean Mass) | 42.39 ± 5.61 | | 41.81 ± 5.25 | | 0.48 | | 41.12 ± 4.79 | | 41.28 ± 5.27 | | 0.82 | |  |
| Smoking During Pregnancy (%)  (yes/no) | 45.7 | | 62.5 | | 0.03 | | 53.2 | | 46.2 | | 0.38 | |  |
| **Males** | | | | | | | | | | | | |  |
| **Number of Participants** | 70 | | 105 | |  | | 92 | | 133 | |  | |  |
| **Demographics/SES** |  | |  | |  | |  | |  | |  | |  |
| Mean Age (range) | 175.3 ± 20.96  (146-227) | | 176.8 ± 22.57  (146-226) | | 0.66 | | 184.0 ± 22.01  (146-227) | | 179.3 ± 21.10  (145-225) | | 0.11 | |  |
| Mean Family Income | 37647.06  ± 13507.1 | | 34904.76  ± 13675.2 | | 0.20 | | 76684.78  ± 10224.9 | | 74812.03  ± 10878.2 | | 0.19 | |  |
| **Parental Education (%)** | M | F | M | F | M | F | M | F | M | F | M | F |  |
| *Some primary school* | 0.00 | 0.00 | 0.00 | 0.00 | 0.50 | .001 | 1.09 | 1.09 | 0.00 | 0.00 | 0.76 | 0.11 |  |
| *Primary school* | 0.00 | 0.00 | 0.00 | 0.00 |  |  | 0.00 | 0.00 | 0.00 | 0.00 |  |  |  |
| *Some high school* | 23.5 | 27.9 | 29.5 | 31.4 |  |  | 9.78 | 6.52 | 6.11 | 4.58 |  |  |  |
| *High school* | 45.6 | 47.1 | 40.0 | 44.8 |  |  | 23.9 | 21.7 | 33.6 | 41.2 |  |  |  |
| *Some college* | 5.88 | 5.88 | 8.57 | 1.90 |  |  | 9.78 | 12.0 | 10.7 | 10.7 |  |  |  |
| *College* | 16.2 | 5.88 | 18.1 | 21.0 |  |  | 30.4 | 20.7 | 26.0 | 18.3 |  |  |  |
| *Some university* | 4.41 | 7.35 | 3.81 | 0.95 |  |  | 4.35 | 9.78 | 4.58 | 6.87 |  |  |  |
| *Bachelor’s degree* | 2.94 | 2.94 | 0.00 | 0.00 |  |  | 16.3 | 17.4 | 13.7 | 14.5 |  |  |  |
| *Graduate degree* | 1.47 | 2.94 | 0.00 | 0.00 |  |  | 4.35 | 10.9 | 5.34 | 3.82 |  |  |  |
| **Physical Characteristics** |  | |  | |  | |  | |  | |  | |  |
| Cortical Thickness | 2.58 ± 0.12 | | 2.56 ± 0.12 | | 0.31 | | 2.54 ± 0.12 | | 2.55 ± 0.11 | | 0.38 | |  |
| Pubertal Stage  (Tanner Scale) | 3.32 ± 0.85 | | 3.36 ± 0.88 | | 0.81 | | 3.51 ± 0.90 | | 3.31 ± 0.87 | | 0.09 | |  |
| Contraceptive Use  (yes/no) | NA | | NA | | NA | | NA | | NA | | NA | |  |
| Height (cm) | 164.27 ± 11.14 | | 166.16± 11.33 | | 0.28 | | 170.23 ± 9.09 | | 166.62 ± 10.59 | | 0.006 | |  |
| Weight (kg) | 58.56 ± 16.49 | | 60.59 ± 17.40 | | 0.44 | | 65.74 ± 17.62 | | 61.52 ± 17.49 | | 0.08 | |  |
| BMI | 21.45 ± 4.68 | | 21.62 ± 4.68 | | 0.81 | | 22.44 ± 4.62 | | 21.89 ± 4.53 | | 0.38 | |  |
| Fat Mass | 9.90 ± 7.69 | | 10.92 ± 8.04 | | 0.41 | | 11.26 ± 8.93 | | 10.60 ± 9.25 | | 0.60 | |  |
| Fat Free Mass (Lean Mass) | 48.65 ± 10.96 | | 50.40 ± 11.06 | | 0.32 | | 54.59 ± 10.71 | | 51.11 ± 10.96 | | 0.02 | |  |
| Smoking During Pregnancy (%) (yes/no) | 27.9 | | 53.3 | | 0.002 | | 51.1 | | 36.1 | | 0.38 | |  |
| Legend: M =Mothers, F=Fathers.  ^1^ All p-values were calculated using Mann Whitney U test unless otherwise specified.  ^2^ Mean household income in this study was $58,879.85 ($56,000 for a 4-person household). Based on the 2006 census, the median family income in the province of Quebec was $58,678 (Statistics Canada, 2006 Census of Population, Statistics Canada Catalogue no. 97-563-XCB2006071).  ^3^ Chi Squared test (or Fisher’s Exact when appropriate) was used to generate p-values | | | | | | | | | | | | |  |

Supplementary Table 2: Cortical Region Parcellation and Gene Expression

| **Cortical Region** | ***NR3C1* Expression** | ***AR* Expression** |
| --- | --- | --- |
| Banks of superior temporal sulcus | 9.501 | 3.343 |
| Caudal anterior cingulate | 9.152 | 2.939 |
| Caudal middle frontal | 9.500 | 3.295 |
| Cuneus | 9.707 | 3.402 |
| Entorhinal | 8.865 | 2.881 |
| Fusiform | 9.156 | 2.963 |
| Inferior parietal | 9.543 | 3.270 |
| Inferior temporal | 9.216 | 3.099 |
| Isthmus cingulate | 9.565 | 3.128 |
| Lateral occipital | 9.595 | 3.221 |
| Lateral orbitofrontal | 9.432 | 3.055 |
| Lingual | 9.782 | 3.325 |
| Medial orbitofrontal | 9.191 | 3.010 |
| Middle temporal | 9.311 | 3.128 |
| Parahippocampal | 9.157 | 2.802 |
| Paracentral | 9.501 | 3.492 |
| Pars opercularis | 9.346 | 3.078 |
| Pars orbitalis | 9.530 | 3.231 |
| Pars triangularis | 9.258 | 3.190 |
| Pericalcarine | 9.651 | 3.478 |
| Postcentral | 9.509 | 3.602 |
| Posterior cingulate | 9.364 | 3.215 |
| Precentral | 9.441 | 3.405 |
| Precuneus | 9.554 | 3.413 |
| Rostral anterior cingulate | 9.048 | 3.030 |
| Rostral middle frontal | 9.416 | 3.112 |
| Superior frontal | 9.352 | 3.205 |
| Superior parietal | 9.488 | 3.363 |
| Superior temporal | 9.280 | 3.233 |
| Supramarginal | 9.432 | 3.257 |
| Frontal pole | 9.357 | 3.224 |
| Temporal pole | 8.883 | 3.025 |
| Transverse temporal | 9.531 | 3.331 |
| Insula | 9.244 | 2.853 |

**Supplementary Table 3: Adjusted Cortical Thickness Models^1^**

| **Group** | **Interaction** | **DF1** | **DF2** | **F Value** | **p** |
| --- | --- | --- | --- | --- | --- |
| **Adjusted Mean Cortical Thickness (sex stratified)** | | | | | |
| Females | Age*Inequality*Income | 1 | 399 | 8.91 | 0.006 |
| Males | Age*Inequality*Income | 1 | 394 | 0.01 | 0.90 |
| **Adjusted Mean Cortical Thickness (sex/income group stratified)** | | | | | |
| Females: Low Income | Age*Inequality | 1 | 189 | 6.98 | 0.03 |
| Females: High Income | Age*Inequality | 1 | 210 | 2.19 | 0.28 |
| Males: Low Income | Age*Inequality | 1 | 173 | 0.12 | 0.84 |
| Males: High Income | Age*Inequality | 1 | 223 | 0.04 | 0.84 |
| **Adjusted Regional Cortical Thickness by Age Correlation Fisher-Z Transformed (sex/income group stratified)** | | | | | |
| Females: Low Income | Inequality*NR3C1 | 1 | 32 | 11.59 | 0.005 |
| Females: High Income | Inequality*NR3C1 | 1 | 32 | 4.18 | 0.090 |
| Males: Low Income | Inequality*NR3C1 | 1 | 32 | 0.02 | 0.88 |
| Males: High Income | Inequality*NR3C1 | 1 | 32 | 0.11 | 0.88 |
| Females: Low Income | Inequality*AR | 1 | 32 | 9.60 | 0.01 |
| Females: High Income | Inequality*AR | 1 | 32 | 2.46 | 0.24 |
| Males: Low Income | Inequality*AR | 1 | 32 | 0.006 | 0.94 |
| Males: High Income | Inequality*AR | 1 | 32 | 0.10 | 0.93 |
| ^1^All models are run after adjusting either mean cortical thickness or regional cortical thickness for presence of Age^2^, Age^2^*Inequality, maternal smoking during pregnancy (yes/no), maternal education level, and paternal education level. Education is measured on the same 9 level scale as in Supplementary Table 1. All p-values are FDR corrected. | | | | | |

**Supplementary Table 4: Adjusted Cortical Thickness Subgroup Analysis^1^**

| **Group** | **Correlated Variable** | **R^2^** | **p** |
| --- | --- | --- | --- |
| **Adjusted Mean Cortical Thickness (sex stratified)** | | | |
| Females | Age | 0.10 | <0.0001 |
| Males | Age | 0.31 | <0.0001 |
| **Adjusted Mean Cortical Thickness (sex/income group stratified)** | | | |
| Females: Low-Income High-Inequality | Age | 0.24 | <0.0001 |
| Females: Low-Income Low-Inequality | Age | 0.04 | 0.06 |
| Females: High-Income High-Inequality | Age | 0.02 | 0.22 |
| Females: High-Income Low-Inequality | Age | 0.14 | <0.0001 |
| Males: Low-Income High-Inequality | Age | 0.31 | <0.0001 |
| Males: Low-Income Low-Inequality | Age | 0.39 | <0.0001 |
| Males: High-Income High-Inequality | Age | 0.26 | <0.0001 |
| Males: High-Income Low-Inequality | Age | 0.27 | <0.0001 |
| **Adjusted Regional Cortical Thickness by Age Correlation Fisher-Z Transformed (sex/income group stratified)** | | | |
| Females: Low-Income High-Inequality | NR3C1 | 0.40 | 0.0002 |
| Females: Low-Income Low-Inequality | NR3C1 | 0.004 | 0.72 |
| Females: High-Income High-Inequality | NR3C1 | 0.18 | 0.02 |
| Females: High-Income Low-Inequality | NR3C1 | 0.37 | 0.0003 |
| Males: Low-Income High-Inequality | NR3C1 | 0.42 | 0.0002 |
| Males: Low-Income Low-Inequality | NR3C1 | 0.38 | 0.0002 |
| Males: High-Income High-Inequality | NR3C1 | 0.31 | 0.0009 |
| Males: High-Income Low-Inequality | NR3C1 | 0.34 | 0.0005 |
| Females: Low-Income High-Inequality | AR | 0.25 | 0.01 |
| Females: Low-Income Low-Inequality | AR | 0.02 | 0.48 |
| Females: High-Income High-Inequality | AR | 0.03 | 0.37 |
| Females: High-Income Low-Inequality | AR | 0.17 | 0.02 |
| Males: Low-Income High-Inequality | AR | 0.24 | 0.01 |
| Males: Low-Income Low-Inequality | AR | 0.19 | 0.02 |
| Males: High-Income High-Inequality | AR | 0.19 | 0.02 |
| Males: High-Income Low-Inequality | AR | 0.18 | 0.02 |
| ^1^Mean cortical thickness or regional cortical thickness were adjusted for presence of maternal smoking during pregnancy (yes/no), maternal education level, and paternal education level. Education is measured on the same 9 level scale as in Supplementary Table 1. | | | |

**Supplementary Figure 1.**

**Supplementary Figure 2. Income and Cortical Thickness by Age.**

**Supplementary Figure 3. General Intelligence (Full-scale IQ by WISC-III) and Cortical Thickness by Age.**
